# Eyeing the Cyr61/CTGF/NOV (CCN) group of genes in development and diseases: highlights of their structural likenesses and functional dissimilarities

**DOI:** 10.1186/s40246-015-0046-y

**Published:** 2015-09-23

**Authors:** Izabela Krupska, Elspeth A. Bruford, Brahim Chaqour

**Affiliations:** Department of Cell Biology, Downstate Medical Center, Brooklyn, NY 11203 USA; HUGO Gene Nomenclature Committee, European Molecular Biology Laboratory, European Bioinformatics Institute, Wellcome Genome Campus, Hinxton, Cambridge CB10 1SD UK; Department of Ophthalmology, Downstate Medical Center, Brooklyn, NY 11203 USA; State University of New York (SUNY) Eye Institute Downstate Medical Center, 450 Clarkson Avenue, MSC 5, Brooklyn, NY 11203 USA

## Abstract

“CCN” is an acronym referring to the first letter of each of the first three members of this original group of mammalian functionally and phylogenetically distinct extracellular matrix (ECM) proteins [i.e., cysteine-rich 61 (CYR61), connective tissue growth factor (CTGF), and nephroblastoma-overexpressed (NOV)]. Although “CCN” genes are unlikely to have arisen from a common ancestral gene, their encoded proteins share multimodular structures in which most cysteine residues are strictly conserved in their positions within several structural motifs. The CCN genes can be subdivided into members developmentally indispensable for embryonic viability (e.g., CCN1, 2 and 5), each assuming unique tissue-specific functions, and members not essential for embryonic development (e.g., CCN3, 4 and 6), probably due to a balance of functional redundancy and specialization during evolution. The temporo-spatial regulation of the CCN genes and the structural information contained within the sequences of their encoded proteins reflect diversity in their context and tissue-specific functions. Genetic association studies and experimental anomalies, replicated in various animal models, have shown that altered CCN gene structure or expression is associated with “injury” stimuli—whether mechanical (e.g., trauma, shear stress) or chemical (e.g., ischemia, hyperglycemia, hyperlipidemia, inflammation). Consequently, increased organ-specific susceptibility to structural damages ensues. These data underscore the critical functions of CCN proteins in the dynamics of tissue repair and regeneration and in the compensatory responses preceding organ failure. A better understanding of the regulation and mode of action of each CCN member will be useful in developing specific gain- or loss-of-function strategies for therapeutic purposes.

## Introduction

The CCN proteins belong to a group of extracellular matrix (ECM)-associated proteins exhibiting a regulatory rather than structural role in the extracellular environment. As such, these molecules have been classified as members of the matricellular protein family which also includes thrombospondins 1 and 2, osteopontin, tenascins C and X, and the SPARC (secreted protein acidic and rich in cysteine)-related proteins, SC1 (SPARCL1) and QR1 [1]. Overall, matricellular proteins are composed of modular motifs found in other ECM proteins. They are different from traditional adhesive proteins such as fibronectin, laminins, and fibrillar collagens, all of which contribute to the structural stability and scaffolding of the tissues [2]. All CCN proteins are expressed in a variety of organisms, and their expression is spatially and temporally regulated during development. In adults, the CCN genes are differentially regulated in tissues that undergo consistent turnover or at sites of injury, repair, and disease [3]. The encoded CCN proteins regulate cell migration, proliferation, cell lineage commitment and tissue specification, morphogenesis, and/or angiogenesis. Several CCN genes are required for embryonic viability although each assumes distinct functions in different tissues and cell types. Matricellular proteins other than CCNs perform similar functions and are co-expressed when tissues undergo events that dictate changes in cell-matrix or cell-cell contact [4]. Yet, mice with homozygous-null mutations in the thrombospondin 1 *(Thbs1),* secreted phosphoprotein 1 *(Spp1)*, and tenascin C *(Tnc)* genes are born with no obvious abnormalities, and only some minor anatomical defects (e.g., kinked tail) emerged during postnatal development. Such an anatomical abnormality might be the result of a defect in collagen fibrillogenesis, which is disordered in the *Thbs2*-null and *Sparc*-null mice [5]. Interestingly, the CCN proteins do not play a role in ECM protein organization even though they assume other essential functions in cardiovascular and skeletal development and pathology. Here, we review the key structural and functional attributes of each of the CCN molecules and their relevance in development and diseases.

### CCN nomenclature

Although the acronym “CCN” refers to the initials of the first three members of this group of genes [i.e., cysteine-rich 61 (CYR61), connective tissue growth factor (CTGF), and nephroblastoma overexpressed (NOV)], there are three additional related members named Wnt-inducible secreted proteins WISP1, WISP2, and WISP3 based on their induction by Wnt ligands. The acronym CCN followed by a number reflecting the chronology of the discovery of these proteins, as shown in Table 1, has been proposed to unify the disparate names used in publications for this group of proteins and genes in different species [6]. For instance, CCN1 was referred to as Cyr61 in mouse [7], GIG1 in humans, and CEF10 in chicken [8], CTGF was referred to as Nov2 in human and fisp12 [9] in mouse, and WISP1 and 2 were sometimes referred to as ELM1 [10] and COP1 [11], respectively. An attempt by Baxter and Twigg to reclassify these proteins as insulin-like growth factor binding proteins (IGFBPs) was not followed as this classification had neither a functional nor structural basis [12]. Indeed, these proteins have no real IGF binding properties and the affinity of some of them to IGF is 1000-fold poorer than the original IGFBPs [13]. For the same reasons, the use of other appellations such as IGFBP-related proteins (IGFBP-rP) was not deemed appropriate. A phylogenetic analysis by Vilmos et al. further supported the concept that the CCN and IGFBP proteins are separate sets of genes [14].

**Table 1:** Nomenclature and chromosomal location of the genes encoding CCN proteins in the human and mouse genome

Unified nomenclature	Approved gene symbol	Approved gene name	Human		Mouse	
			Chromosome	Chromosome location	Chromosome	Chromosome location
CCN1	CYR61	Cysteine-rich, angiogenic inducer, 61	1	1p22.3	3	70.18 cM
CCN2	CTGF	Connective tissue growth factor	6	6q23.2	10	11.84 cM
CCN3	NOV	Nephroblastoma overexpressed	8	8q24.12	15	21.49 cM
CCN4	WISP1	WNT1 inducible signaling pathway protein1	8	8q24.22	15	29.3 cM
CCN5	WISP2	WNT1 inducible signaling pathway protein 2	20	20q13.12	2	84.49 cM
CCN6	WISP3	WNT1 inducible signaling pathway protein 3	6	6q21	10	20.19 cM

Murine loci are expressed in centimorgans (CM)

The unified CCN nomenclature, which has been endorsed by the International CCN Society (http://www.ccnsociety.com), emphasizes the structural relatedness of these molecules and also eliminates any confusion from the multiple names that have previously been given to them. However, note that the root symbol CCN is already in use and approved by the HUGO Gene Nomenclature Committee (HGNC) for the cyclin gene family, and the original gene symbols (*CYR61*, *CTGF*, *NOV*, *WISP1*/*2*/*3*) as listed in Table 1 and at the HGNC’s “CYR61/CTGF/NOV matricellular proteins” webpage (http://www.genenames.org/cgi-bin/genefamilies/set/1046) are still widely used by many investigators. While the CCN nomenclature is increasingly adopted and accepted in the literature, and will be used hereafter in the present review, the approved gene symbols will be used when a specific gene is explicitly discussed.

Interestingly, the CCN genes can further be subdivided into members that are indispensable for embryonic viability (i.e., CCN1, CCN2, and CCN5), and other members that are not critical for embryonic development (i.e., CCN3, 4 and 6) as deficiency in mice of these proteins did not compromise fetal development and resulted in a superficially mild or no phenotype [15–17]. Nevertheless, the classification of the CCN proteins as matricellular proteins has held up well, since published data so far continue to lend credence to their matricellular membership and classification. Of note, the matricellular proteins—not included in the CCN family—now include other proteins such as periostin, R-spondins, and the short fibulins including hemicentin, galectins, small leucine-rich proteoglycans, and autotaxin [4]. These proteins are also structurally and functionally diverse, and none of them recapitulate the biological functions of the CCN proteins.

### CCN gene and protein structure

**Fig. 1:**
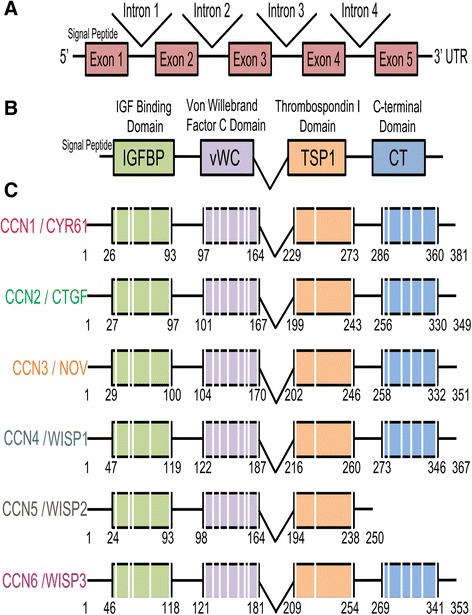
CCN gene and protein organization. Schematic diagrams of the overall gene (**a**) and protein domain organization (**b**) showing the secretory signal and the homologous domains (*IGFBP* insulin-like growth factor binding protein, *VWC* von Willebrand factor type C repeat, *TSP-1* thrombospondin type 1 repeat, *CT* carboxy-terminal domain). The specific modular organization of each member of this group of genes is shown in **c**. Not to scale

The primary structure of the CCN genes contains a common intron-exon pattern with most of the family members containing five exons interspersed by four introns (Fig. 1). The gene structure can be further divided into the N-terminus encoded by the first, second, and third exons and the C-terminus encoded by the fourth and fifth exons. The N- and C-termini are separated by a variable hinge region that is sensitive to proteolysis. The first exon corresponds to the signal peptide sequence, followed by four exons that correspond to the four conserved domains with sequence homologies to insulin-like growth factor binding proteins (IGFBPs), von Willebrand factor type C repeat (vWC), thrombospondin type 1 repeat (TSP-1), and a carboxyl domain (CT) that contains a cysteine knot motif [18, 19] present in growth factors including transforming growth factor β (TGFB1), nerve growth factor (NGF), platelet-derived growth factor (PDGFA), and human chorionic gonadotropin [20]. The CT domain is believed to be involved in dimerization and heparin binding [14]. The exception to this shared gene structure is CCN5/*WISP2*, which lacks the last exon encoding the fourth CT module and contains only four exons.

**Fig. 2:**
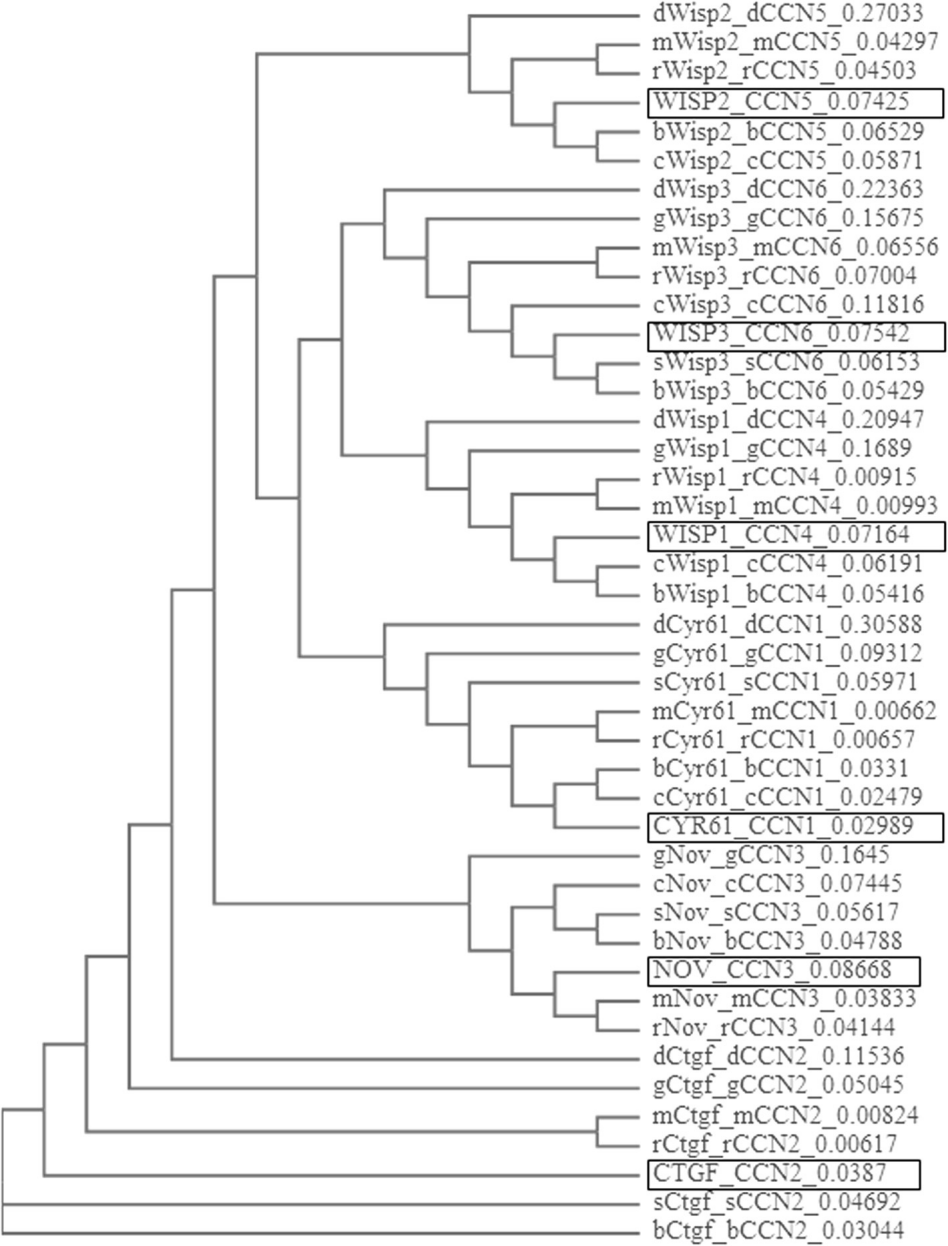
Dendrogram showing the phylogenetic relations between the genes encoding the CCN proteins. The total number of differences between sequences (i.e., probability of relatedness) is shown to the right. The human genes are framed in the diagram. *m* mus musculus-mouse, *r* rattus norvegicus-rat, *g* gallus gallus-chicken, *b* bos taurus-cow, *s* sus scrofa-pig, *c* canis lupus familiaris-dog, *d* danio rerio-zebrafish

According to Vilmos et al., CCN genes emerged after the divergence of vertebrates and invertebrates [14] and have been characterized from human, mouse, rat, pig, cow, chicken, quail, and frog (Fig. 2). Zebrafish, which is partially tetraploid teleost and possesses orthologous pairs of genes that are found as single copies in mammals, expresses nine genes that have strong homology to the mammalian CCNs; three are paralogs of CCN1, two of CCN2, two of CCN4, one of CCN5, and one of CCN6 [21]. No paralog of CCN3 was found. Their tetra-modular pattern and the shared identity with functional domains of other regulatory proteins suggest that the CCN genes are products of exon shuffling during evolution when multicellular organisms were formed. In this evolutionary process, other recombination mechanisms have also occurred including DNA duplication, deletion, inversion, conversion, and translocation [22]. The evolutionary exon shuffling generated multimodular molecules with four conserved domains and disulfide linkage patterns [23].

**Fig. 3:**
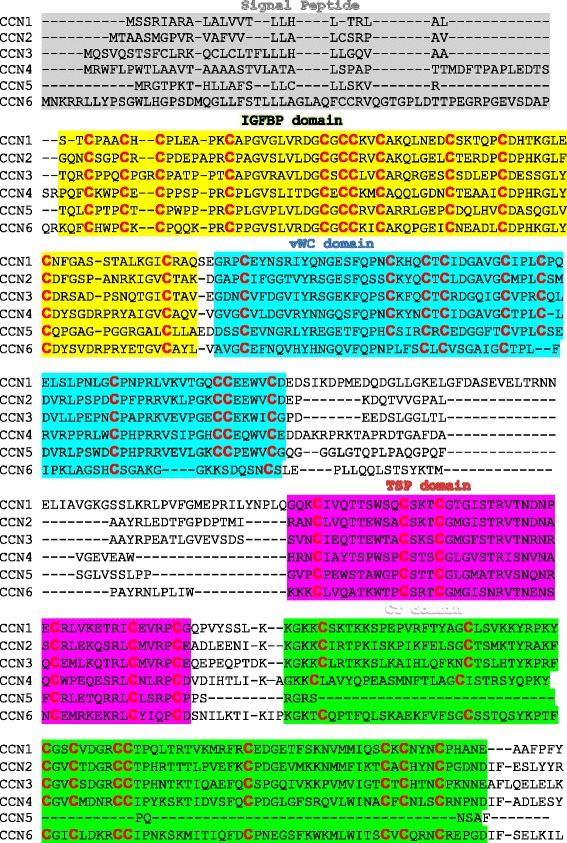
Sequence alignments of the CCN proteins. Human sequence alignment was performed using the ClustalW2 multiple sequence alignment program. The sections of the sequences corresponding to each domain are highlighted using a different color. CCN proteins share 38 conserved cysteine residues (in *red*). The exceptions are CCN5 and CCN6, which contain 28 and 34 cysteine residues, respectively

The encoded CCN proteins share about 40–60 % similarity in their amino acid sequences including a series of 38 cysteine residues that are strictly conserved in their position and number and form 17 disulfide bonds spread throughout the entire primary sequence of these molecules [19] (Fig. 3). The exceptions are CCN5, which contains only 28 conserved cysteines, and CCN6, which lacks 4 cysteines in the second module [24]. The recombinant remodeling allowed the four domains of each CCN protein to fold into unique three-dimensional structures dictated by the different electrostatic charges on their surfaces. The latter defined their 3D conformation, their interactivity with other molecules, and their function. Interestingly, the relatively long linker region between the vWC and TSP1 domains and the shorter linkers between IGFBP and vWC and between TSP1 and CT provide both an overall flexibility of the full-length molecule and a local rigidity within the molecule, with the IGFBP and CT domains (at the extreme N- and C-terminal ends, respectively) cocked in almost opposite directions. As shown in recent studies of CCN1, proteolytic cleavage of the protein produces truncated forms of the molecule in which amino acid residues hidden in the compact structure of the intact protein become exposed and readily accessible to receptors and interacting partners [25].

### CCN genes in development and diseases

#### CCN1/*CYR61*

The human *CYR61* gene encoding CCN1 was mapped to chromosome 1 at band p22.3 (Table 1) [26, 27]. Genetic studies have suggested a relationship between a missense mutation of the *CYR61* gene and patients with severe atrial septal defects [16, 28]. The mutation leads to an exchange of residues with different properties in a highly conserved position in the N-terminal IGFBP module of CCN1. Consistent with these observations, conventional deletion of the *Cyr61* gene in mice caused severe atrioventricular septal defects (AVSD) [16]. AVSDs are characterized by the complete or partial absence of partitioning of the atrioventricular valve (AV) and are common genetic disorders that cause congenital heart disease in humans. CCN1-deficient mice are characterized by early onset of apoptosis in the AV cushion that likely hampers successful fusion between the cushion tissue and the atrial septum. Thus, *CYR61* gene sequence screening may be important in genetic analyses of patients with congenital heart diseases.

During embryonic development, CCN1 is widely expressed in the cardiovascular system [9]. Prominent cardiac expression of CCN1 was seen as early as E8.5 in mice expressing lacZ under the control of the endogenous *Cyr61* promoter. CCN1 expression continued at E10.5 especially in the trunk arteriosus, which later divides to form the aorta and pulmonary trunks [16]. CCN1 expression was also found in all major arteries branching from the heart of the developing fetal circulatory system. These include the aortic and pulmonary trunks which are branches of the aortic arches and the dorsal aorta and the umbilical artery which are of mesodermal origin. The generation of CCN1-deficient mice provided some insights into the vital role of CCN1 during cardiovascular development. A large majority of CCN1 null embryos died of hemorrhage and/or placental defects between E11.5 and E14.5, while a fraction of the embryos died earlier as a result of defects in chorioallantoic fusion [29]. Vascular defects seemed to originate either at the chorioallantoic junction, where the allantois failed to fuse with the placenta resulting in severe undervascularization in the placental labyrinth, or from defects of large vessel bifurcation resulting in a poor vascular coverage of the chorionic plate. Large fetal vessels such as the aorta appeared dilated, similar to large aneurysms. Vascular cells such as endothelial and smooth muscle cells were not confined within their layers and were mislocalized in the media layer of the aorta. Similarly, endothelial-specific deletion of *Cyr61* in mice resulted in the formation of vessels with a large diameter and loss of vessel organization into the arteries, capillaries, and veins [30]. Subsequently, CCN1-deficient embryos suffered from compromised integrity and hemorrhage of arterial vessels and capillaries.

A *Cyr61* promoter-driven reporter gene expressed in transgenic mice further detected *Cyr61* gene expression in a wide range of other tissues including the respiratory system, the embryonic skeletal system including the notochord, sclerotomes, limbs, and developing bone, the developing nervous system including the ventral spinal cord, dorsal root ganglia, parts of the mesencephalon and telencephalon, and the olfactory bulb, the embryonic epidermis, and the inner root sheath of hair follicles [7]. The tissue-specific function of CCN1 during embryonic development has not been established although several laboratories including ours are currently using the Cre-lox system in mice to determine the functional relevance of CCN1 expression in different tissues and cells [30]. At the molecular level, the CCN1 protein seems to act as an adaptor or scaffold that can bring cytokines and growth factors into close proximity to the cell surface by binding integrins, heparan sulfate proteoglycans, and receptor tyrosine kinases [31–33]. Together with the known adhesive and chemotactic functions of the recombinant protein, CCN1 may promote cell migration and guidance during tissue patterning and differentiation. The CCN1-integrin interaction in various cell types activates the RAS signaling pathway axis including MAPK1 and/or AKT1, leading to gene expression of key regulatory proteins that promote cell cycle progression, adhesion and tube formation [34–38]. In addition, CCN1 binds directly to the low-density lipoprotein (LDL) receptor proteins (LRPs) and upregulates Wnt signaling, which activates transcription of targeted genes [39]. Collectively, CCN1 signaling culminates in the upregulation of a broad spectrum of genes that modulate cell growth and differentiation and connective tissue remodeling (e.g., matrix metalloproteinases (MMPs), tissue inhibitor of metalloproteinases (TIMPs), integrins, and VEGF) [40–42].

In the adult, genome-wide scans and association studies have established that the *CYR61* gene showed the third highest mean fold expression differences in a group of obese men with metabolic syndrome as compared with controls [43]. Bouchard et al. further identified up to five polymorphisms within the *CYR61* gene regulatory region and directly linked them to the levels of plasma LDL and HDL cholesterol in a cohort of ~700 obese individuals comprising both men and women [44]. Meanwhile, Schutze et al. further identified a CA polymorphic repeat within the *CYR61* promoter, characteristic of disorders affecting bone metabolism [45]. These observations provide a genetic argument in favor of the involvement of *CYR61* gene regulation in inflammatory disorders. In support of this hypothesis, experimental and clinical data showed that the *CYR61* gene is particularly highly expressed at sites of inflammation and tissue repair. Increased CCN1 levels have been found in a number of chronic inflammatory diseases including colitis [46], rheumatoid arthritis [47], and atherosclerosis [48, 49]. Moreover, analyses of fluids and tissue biopsies from human clinical specimens have shown increased levels of CCN1 in several ocular vascular complications, including proliferative diabetic retinopathy (PDR) and active ophthalmopathy [50, 51]. Similarly, the levels of CCN1 increased in retinal blood vessels in the early stages of diabetes and in late stages of proliferative disorders in mouse models of diabetic and ischemic angiopathies [52, 53]. Whether CCN1 expression under pathological conditions recapitulates biological events characteristic of earlier developmental stages, as many fetal genes do, is unknown. Interestingly, CCN1 expression has been found to be downregulated during tissue involution, in avascular tissues, and under conditions associated with vasoobliteration, which is consistent with a potential role of this protein in vessel formation, stabilization, and integrity [54–56]. In the mouse model of oxygen-induced retinopathy, CCN1 enhanced physiological adaptation of the retinal vasculature to hyperoxia and reduced pathological angiogenesis following ischemia [57]. However, CCN1 protein is a substrate for numerous proteases that are highly expressed and released at sites of inflammation [58]. In particular, vitreal fluid samples from patients with PDR contained CCN1 fragments recognizable by specific antibodies to IGFBP and vWC domains, and only a few samples exhibited an additional product that was also detectable by TSP1-specific antibodies [25]. The truncated forms containing either the partial or complete two-module IGFBP-vWC portion were predominantly represented in PDR fluids along with other proangiogenic and permeability factors such as VEGF and MMP2. Whether and how proteolytic events affect CCN1 function, and whether this defines new cellular responses, is yet to be elucidated.

#### CCN2/*CTGF*

The *CTGF* gene encoding CCN2 maps to human chromosome 6q23.1 [59] and to the A3-B1 region of murine chromosome 10 (Table 1) [60]. It is located within a commonly deleted segment of chromosome 6 (q22.1–23.2) in biopsies from patients with abdominal aortic aneurysm (AAA) [61]. Acquired chromosomal aberrations associated with retrotransposon propagation at this location predispose to sporadic AAA. In addition, genes located in this chromosomal region have been implicated in the development of systemic sclerosis and hepatic fibrosis (HF) [62, 63]. Functional single nucleotide polymorphisms (SNPs, rs6918698) implicated CCN2 as a major actor in severe HF in schistosome-infected Chinese, Sudanese, and Brazilian subjects [64]. Another study further confirmed that one *CTGF* gene polymorphism (rs6918698; −945 G/C) is a significant risk factor for the progression of hepatitis C-related chronic liver diseases [65].

Although congenital diseases have, thus far, not been mapped to genetic alterations of the *CTGF* gene, its expression plays an important role in normal embryonic development. The spatial and temporal expression of CCN2 during development is similar to that of CCN1, with high levels present in endothelium and mural cells of the blood vessels, heart, and skeletal tissues such as the cartilage and maturing chondrocytes [66]. This pattern of CCN2 expression during development appears to be largely conserved in vertebrates including fish, frog, and mouse. A loss-of-CCN2 function in zebrafish showed developmental delays and distortion of the notochord [67]. Homozygous *Ctgf* mutant mice were found to have short, misaligned, and inward-bent sterna and kinked ribs, possibly due to decreased growth plate angiogenesis, chondrocyte migration, and matrix degradation, resulting in a visibly reduced thoracic volume [15]. Perinatal lethality ensues as the *Ctgf* mutant mice showed signs of cyanosis and difficulty to breathe, continuously gasping for air. These are the characteristics of numerous human chondrodystrophic pulmonary hypoplasias that frequently prove lethal soon after birth [68]. The lungs of *Ctgf* mutant mice were hypoplastic and seemingly arrested at the canalicular stage of development, with reduced cell proliferation and increased apoptosis [69]. Reduction in lamellar body size in type II pneumocytes, coupled with the great reduction of available alveolar space in the mutant mouse lungs, suggests reduced surfactant production and delayed lung maturity.

In *Ctgf* knockout mice, vascular and skeletal defects begin to emerge at later stages of development [70]. Large vessels in *Ctgf*-null embryos show defects in the organization of tunica media where smooth muscle cells appear less spindle-like, more heterogeneous in size, and not organized into distinct layers when compared to wild-type embryos. In addition, the microvascular endothelium in *Ctgf*-null mice is incompletely covered by pericytes. Loss of CCN2 leads also to diminished expression of vessel maturation markers, such as angiopoietin 1, and elevated expression of immature vasculature markers, such as versican. Furthermore, *Ctgf*-deficient mice exhibit defects in endothelial basement membrane organization where expression of fibronectin and its association with blood vessels is significantly decreased, subsequently leading to discontinuous incorporation of type IV collagen in the basement membrane of the microvasculature. In endothelial cells, CCN2 mediates cell adhesion, directional migration, and proliferation through integrin αvβ3 [71]. CCN2 is also a ligand for integrins α5β1 and α6β1 that are required for endothelial basement membrane formation and vessel stabilization in vitro [72, 73]. However, besides interacting with multiple integrins, CCN2 also modulates the activities of several other ECM components including VEGF, TGF-β, and Wnt signaling [19]. CCN2 has been reported to bind VEGF-A^165^ at two sites through its TSP1 and CT domains [74]. In addition, the TSP1 domain of CCN2 binds to LRP1, which acts as a co-repressor of the Frizzled receptor and downregulates Wnt signaling [75, 76].

In the adult, *Ctgf* mRNA is expressed at high levels in the spleen, ovary, gastrointestinal tract, prostate, heart, and testis and at lower levels in the thymus, placenta, lung, skeletal muscle, kidney, and pancreas [77]. No expression of CCN2 has been detected in the brain, liver, and peripheral leucocytes. Studies of diseased tissues from human clinical specimens and animal models established a correlation between high levels of the CCN2 protein and excessive accumulation and deposition of ECM proteins in fibrotic tissues, suggesting a pathogenic role for CCN2 in fibroproliferative disorders [78]. However, transgenic models of CCN2 overproduction in a number of tissues exhibited various phenotypes, including no fibrotic reaction, mild fibrosis, and clear fibrotic phenotypes depending on the CCN2 levels [79]. Specific thresholds of CCN2 levels are required to induce cell type-specific effects. At the molecular level, CCN2 is able to stimulate the transition of differentiated cells such as tubular epithelial cells, endothelial cells, and fibroblasts to activated myofibroblasts [80–82]. In this transition, proteins that are expressed by differentiated cells, such as E-cadherin in epithelial cells, are lost while proteins expressed by myofibroblasts, such as fibrillar collagens and α-smooth muscle actin, are expressed [83]. There is evidence that anti-CCN2 therapy can successfully attenuate fibrotic reactions in the kidney, liver, and other organs exhibiting fibrotic reactions, suggesting that therapies targeting CCN2 and its signaling pathways may be beneficial for the treatment of the fibrotic diseases [78].

Meanwhile, CCN2 plays an important role in vascular diseases as well. Numerous studies have shown a close correlation in topography and timing between *CTGF* gene expression and spatial distribution and expansion of the blood vessels in response to ischemic injury, as in neovascular diseases of the eye [51]. Examination of the vascular implications of CCN2 expression, or lack thereof, in response to hyperoxic or hyperglycemic injury showed a close correlation between *CTGF* gene expression and blood vessel growth and stabilization [51, 84, 85]. Chintala et al. have shown that CCN2 induces neovascularization through p53-dependent modulation of *MMP2* gene expression, which drives vascular remodeling through degradation of the basement membrane and subsequent formation of new vascular patterns [86]. Interestingly, reduced levels of CCN2 in ischemic retinas correlate with reduced VEGF gradient levels, indicating that coordinated regulation between CCN2 and VEGF indeed exists. CCN2 can bind VEGF and inhibit the angiogenic activity of both molecules when MMPs are not available, forming a negative feedback loop among these angiogenic factors [87].

#### CCN3/*NOV*

CCN3 is encoded by the *NOV* gene which maps to human chromosome 8q24.1. CCN3 is expressed during development in derivatives of all three germ layers, with high levels detected in skeletal muscle, smooth muscle of vessel walls, the nervous system, adrenal cortex, and differentiating chondrocytes [88, 89]. CCN3 seems to play a role during myogenesis and in the formation and/or stabilization of adhesion structures specialized in force transmission from the muscle to tendon [90]. However, CCN3-deficient mice developed normally to adulthood and both the males and females were fertile [91]. Similarly, mutant mice in which the protein had lost the vWC domain were in apparent good health although numerous skeletal defects were observed [92]. In particular, development of the appendicular and axial skeleton was affected with enlarged vertebrae, elongated long bones and digits, delayed ossification, increased bone mineralization, and severe joint malformations. In addition, enlargement and abnormal remodeling of the endocardial cushions, associated with septal defects and delayed fusion, were observed in developing embryos. In adults, cardiomyopathy was apparent, with hypertrophy and calcification of the septum and left ventricle dilation. Muscle atrophy was seen by 5 months of age, associated with transdifferentiation to fat. Premature tissue degeneration was also seen in the lens, with cataracts in 6-month-old mice.

CCN3 transgenic mice, in which CCN3 expression was driven by a 2.3-kb *Col1a1* promoter, showed osteopenia compared with wild-type mice [93]. In addition, bone regeneration in *Nov* knockout mice was accelerated compared with that in wild-type mice. CCN3 acts as a selective negative regulator of the expression of osteoblast-related genes, e.g., *Runx2*, *Sp7*, *Col1a1*, *Alpl*, and *Bglap*, and inhibits osteoblastic differentiation [94]. In addition, CCN3 function has been attributed to its functional and physical interactions with many molecules such as integrin, bone morphogenic proteins (BMPs), and Notch [95]. CCN3 was proposed to fine tune the activity of BMPs during bone regeneration by antagonizing BMP effects on its downstream targets such as Noggin, Chordin, and Gremlin1. BMP inhibition by CCN3 results in the suppression of mesenchymal stem cells differentiating into osteoblasts [39]. Moreover, CCN3 binds to specific epidermal growth factor motifs of the Notch receptor and activates downstream signaling during myogenesis, which underscores the multifaceted activities of this molecule [96].

#### CCN4/*WISP1*

CCN4 was initially published as “Wisp1” because, like *WISP2*/CCN5 and *WISP3*/CCN6, it was induced by WNT1 but not by WNT4 [10]. CCN4 was initially identified in the mouse mammary epithelial cell line C57MG transformed by WNT1 [97]. EST database screening further identified *Wisp1* as homologous to *Nov* [98]. CCN4 expression is restricted to osteoblast and osteoblastic progenitor cells during development and bone fracture repair [99]. CCN4 is expressed in several adult tissues including the epithelium, heart, kidney, lung, pancreas, placenta, ovaries, small intestine, spleen, and brain [100].

CCN4 availability is modulated by interactions with decorin and biglycan, two ECM-associated proteoglycans [99]. In addition, CCN4 potentiates the effect of BMP2 on osteoprogenitor cells in the bone marrow and promotes osteoblastic differentiation while repressing chondrocytic differentiation [101, 102]. CCN4 enhances BMP2 signaling through a mechanism involving binding to integrin α5β1 [102]. This is unlike other CCN proteins like CCN2 and CCN3, which rather inhibit BMP activity in osteoblastic lineage differentiation [103]. CCN4 variant WISP1v lacking the vWC module was reported in a few human gastrointestinal cancer tissues and normal chondrocytes undergoing endochondrial ossification [104]. Indeed, overexpression of WISP1v enhanced the gene expression of alkaline phosphatase, a mineralization marker in chondrocytic cells [105].

Meanwhile, previous studies have identified *WISP1* as an oncogene that accelerates growth and morphological transformation of alveolar epithelial cells and attenuates cell apoptosis in response to DNA damage [106]. CCN4 signaling inhibits cell death following DNA damage by activation of the pro-survival AKT pathway and the antiapoptotic BCL2L1 (Bcl-XL) [107]. Subsequently, mitochondrial release of the cytochrome c and caspase cascade is suppressed. In a similar way, activation of the AKT kinase pathway in alveolar epithelial type II (ATII) cells treated with CCN4 induces a significant increase in cell proliferation, epithelial-mesenchymal transition (EMT), upregulation of gene expression of pro-fibrotic markers, and production of ECM [108]. These effects were confirmed in both the mouse model of pulmonary fibrosis and patients with idiopathic pulmonary fibrosis, which leads to distortion of normal tissue architecture and a loss of lung function [109]. Most importantly, reduction of CCN4 activity by CCN4-specific antibodies resulted in decreased expression of genes characteristic of fibrosis and significantly attenuated the progression of the lung fibrosis suggesting that CCN4 exhibits antifibrotic activities opposing the profibrotic activities of CCN2 [110].

In primary neuronal cells, CCN4 can avert microglial inflammatory cell death during Β-amyloid (Aβ) toxicity and prevent oxidative stress injury [111]. CCN4 promotes neuronal cell survival during oxidative stress by enhancing post-translational phosphorylation of FOXO3a and its sequestration into the cytoplasm of neurons with protein 14-3-3 [112]. CCN4 also minimizes deacetylation of FOXO3a, prevents caspase 1 and 3 activation, and potentiates the neuroprotective potential of SIRT1 activity by preventing SIRT1 caspase degradation. Therefore, CCN4 has a therapeutic potential in fibrotic and neurodegenerative diseases and cancer.

#### CCN5/*WISP2*

CCN5 is structurally unique among the CCN family members because it lacks the carboxy-terminal domain that is responsible for the proliferative activity of CCN1 and CCN2 [17]. This suggests that CCN5 is a naturally occurring dominant negative molecule that modulates the effects of other CCN proteins [113].

CCN5 is expressed in nearly all embryonic stages of development, and some level of CCN5 remains present in multiple organs in adult organisms [114]. This widespread expression pattern together with the unique structure of CCN5 suggests that it performs a diverse range of functions during development. CCN5 is expressed as early as E4.5 at the implantation stage of the blastocyst in the uterine wall and continues to be expressed in all three germ layers: endoderm, mesoderm, and ectoderm. It is believed that the antiproliferative activities of CCN5 counterbalance the mitogenic activities of the other CCN family members [114]. Not surprisingly, CCN5-null mice and transgenic mice overexpressing CCN5 die at or before gastrulation due to improper implantation [115]. CCN5 is closely associated with the cell surface, although its presence is also detected in the nucleus in the cells of multiple adult organs including the kidney, ovary, brain, heart, and lung [116]. Particularly, high levels of CCN5 expression are found in the endothelium and smooth muscle of the veins, arteries, and the myocardium of the heart in early embryonic and adult tissues. CCN5 inhibits the proliferation and migration of the smooth muscle in both cell culture and animal models [117, 118]. Consequently, it prevents invasiveness of cells by decreasing the activities of MMPs [119]. Expression of CCN5 is highly upregulated by estrogen, especially in MCF-7 breast cancer lines that express estrogen receptor alpha (*ESR1*), making it an oncogene [120]. *WISP4* knockdown by sh-CCN5 in MCF-7 breast cancer cells leads to an increased expression of the components of the TGFB1 (TGF-β) signaling pathway and induction of breast cancer invasiveness. TGFB1 plays an important role in tumor progression by promoting angiogenesis, EMT, and metastasis [121]. CCN5 attenuates TGFB1 signaling and, by the same token, suppresses the progression of CCN2-mediated fibrogenesis [122]. Concordantly, TGFB1 signaling and cardiac fibrosis are inhibited in CCN5 transgenic mice in contrast to CCN2 transgenic mice. A study by Yoon et al. further demonstrated that deletion of the C-terminal domain of CCN2 transformed CCN2 into a CCN5-like molecule, whereas fusion of the CT domain of CCN2 to the carboxy-terminus of CCN5 transformed CCN5 into a CCN2-like pro-hypertrophic and pro-fibrotic molecule in cardiomyocytes [122].

#### CCN6/*WISP3*

CCN6 is an essential protein involved in the maintenance of human articular cartilage [123]. Mutations in human *WISP3* cause progressive pseudorheumatoid dysplasia that manifests itself in early childhood with loss of articular cartilage causing multiple joint and bone abnormalities [124]. Surprisingly, no apparent phenotype was caused by CCN6 deficiency in mice, which made it challenging to understand the functional significance of CCN6 in development and disease. Importantly, CCN6 seems to be involved in gene regulation of proteinases, such as the ADAMTSs and MMPs that cause cartilage matrix degradation found in PPD [123, 125]. Overexpression of CCN6 in immortalized chondrocytic cell lines dramatically reduced the expression of ADAMTS4 and ADAMTS5, while the expression of MMP1 and MMP10 was elevated [123]. On the other hand, *WISP3* knockdown in cytokine-stimulated primary chondrocytes resulted in elevated expression of ADAMTS5 and repression of MMP10. These observations suggest that CCN6 is involved in complex context-dependent control of metalloproteinase expression. However, CCN6 was shown to be downregulated in invasive and metastatic breast cancers, suggesting that it also exerts a tumor suppressive function [126]. In the absence of CCN6, there is activation of the BMP4-induced SMAD-independent MAP3K7 (TAK1)/MAPK14 (p38) pathway that promotes invasion [127]. Conversely, when present, CCN6 protein binds to BMP4 and inhibits the BMP4-mediated activation of MAP3K7/MAPK14 kinases and decreases the invasiveness of breast cancer cells [128]. Thus, CCN6 can be considered as a possible therapeutic target for breast and metastatic cancers.

## Conclusion

Taken together, data shows that the CCN proteins regulate development and pathology of different organ systems in various contexts. The requirement of CCN1, CCN2, and CCN5 for embryonic viability indicates their importance in tissue formation, organization, and function. Survival of mice deficient in CCN3, CCN4, or CCN6 suggests that either these molecules are not required for structural integrity and/or function or the existence of a functional redundancy with other molecules. However, all CCN proteins seem to be involved in the physiological and/or pathological responses of tissues and organ systems to injurious stimuli. Whether re-expression of these proteins in disease states recapitulates developmental processes is still unknown. Clearly, the function of all CCN proteins are cell- and tissue-specific and careful consideration of the context in which they are expressed should be taken into consideration when assessing their usefulness as potential markers or therapeutic targets in pathological conditions.
